# Supplementary material for: A metagenomics-based diagnostic approach for central nervous system infections in hospital acute care setting
Source: Sci Rep. 2020 Jul 8;10:11194. doi: 10.1038/s41598-020-68159-z (PMC7343800; doi:10.1038/s41598-020-68159-z)
Supplement: Supplementary file 1 — Supplementary information [file 41598_2020_68159_MOESM1_ESM.pdf]

Title: A Metagenomics – Based Diagnostic Approach for Central Nervous System Infections in Hospital Acute Care Setting

Authors: Mohammad Rubayet Hasan

Sathyavathi Sundararaju

Patrick Tang

Kin-Ming Tsui

Andres Perez Lopez

Mohammad Janahi

Rusung Tan

Peter Tilley

Supplemental table 1: Summary of mNGS analytical data

Group	Sample name	DNA conc. ng/μl	Library Con. (nM)	IDseq run time (hr)	Total reads	QC passed (%)	Total non-host reads	Non-host read (%)	IC read
Training set	NFW	-	-	3.04	35,562	14.35	4,360	12.26	-
	Neg1	0.18	1.59	0.84	3,757,836	20.88	5,214	0.14	1265
	Neg2	0.71	41.79	0.66	19,849,652	46.76	830	0	165
	Neg3	0.18	6.98	0.87	30,351,484	20.12	10,888	0.04	21,640
	Neg4	2.07	30.81	0.64	27,575,002	22.68	528	0	55
	Spike1	0.28	1.24	0.85	5,724,496	33	317,182	5.54	1,231
	Spike2	0.4	2.8	1.94	13,997,484	45.03	998,966	7.14	416
	Spike3	0.33	0.54	1.72	9,346,120	44.56	1,266,608	13.55	621
	Spike4	0.29	1.08	2.98	17,279,180	58.09	5,862,907	33.93	2,480
	Spike5	0.29	0.6	2.57	11,578,754	59.46	4,820,450	41.63	2,887
Validation set	CW001	0.25	13.36	3.06	32,890,856	11.26	2,222	0.01	534
	CW002	0.26	18.5	0.52	10,559,416	20.66	1,820	0.02	580
	CW004	0.18	20.02	0.61	18,099,016	16.28	1,616	0.01	674
	CW005	0.15	9.65	0.79	31,739,360	16.87	3,910	0.01	1,806
	CW006	0.17	3.39	0.86	22,271,984	27.83	11,686	0.05	8,255
	CW007	19.87	18.54	0.75	21,994,266	27.76	30,534	0.14	10
	CW008	0.66	50.85	0.75	18,469,032	44.8	4,332	0.02	73
	CW010	0.18	1.59	3.13	3,757,836	20.88	5,218	0.14	1,425
	CW012	0.17	7.79	0.82	32,219,370	12.34	5,946	0.02	3,001
	CW013	0.82	9.17	10.06	34,654,820	13.08	3,376	0.01	82
	CW015	0.38	21.56	6.31	31,121,416	32.36	4,728	0.02	277
	CW016	0.56	18.89	6.41	36,670,904	20.81	4,544	0.01	787
	CW018	0.21	27.36	0.84	29,259,136	18.62	4,300	0.02	1,215
	CW019	0.18	1.56	1.32	41,306,254	12.01	31,770	0.08	19,058
	CW020	0.17	9.15	1.02	22,245,768	24.17	17,888	0.08	5,598
	CW021	0.23	51.23	2.06	18,393,708	50.04	2,430,956	13.22	19
	CW022	0.24	15.07	0.59	13,064,630	28.79	958	0.01	63
	CW023	0.23	22.6	1.32	17,192,624	33.59	900	0.01	123
	CW024	0.21	16.57	0.47	16,230,074	46.79	12,352	0.08	29
	CW025	0.26	4.71	1.52	44,424,176	7.44	4,440	0.01	3,644
	CW028	0.39	0.86	1.35	23,206,714	22.22	34,010	0.15	24,362
	CW029	0.37	7.75	1.27	26,262,240	11.16	2,356	0.01	269
	CW030	0.4	1.42	0.89	20,129,178	19.73	16,658	0.08	17,403
	CW031	0.17	0.37	0.85	7,287,630	26.48	7,282	0.1	2,922
	CW033	0.35	1	0.67	16,758,066	11.59	21,398	0.13	38,403
	CW034	6.82	32.78	0.85	21,070,728	39.22	142,374	0.68	23

	CW036	2.99	24.9	0.64	28,923,904	46.68	938	0	83
	CW037	3.94	22.5	1.27	23,056,630	33.51	750	0	47
	CW038	0.46	3.61	0.64	20,949,082	25.34	5,838	0.03	8,126
	CW040	0.39	0.6	0.88	28,833,986	19.32	19,046	0.07	42,434
	CW043	0.33	1.08	0.62	25,137,054	10.95	15,460	0.06	32,584
	CW044	0.41	4.25	0.58	16,987,022	17.79	8,062	0.05	12,815
	CW045	0.39	12.05	0.46	18,191,994	28.37	3,028	0.02	3,477
	CW046	0.41	23.14	0.55	20,316,044	30.71	1,480	0.01	1,164
	CW048	0.32	1.33	0.77	20,439,102	18.95	10,778	0.05	20,467
	CW049	0.34	16.9	0.5	11,864,764	26.61	1,070	0.01	939
	CW050	0.31	8.38	0.51	20,596,158	18.67	3,316	0.02	4,133
	CW052	0.41	1.41	0.56	20,716,122	16.82	10,582	0.05	17,467
	CW053	0.34	1.99	0.52	20,271,082	18.46	7,414	0.04	12,671
	CW054	0.33	1.61	0.65	17,283,902	18.71	4,028	0.02	5,941
	CW055	0.35	4.07	0.56	17,592,450	13.07	7,192	0.04	14,684
	CW056	0.3	9.48	0.48	20,395,708	23.49	3,142	0.02	4,139
	CW057	0.31	6.33	0.57	16,962,578	25.76	4,524	0.03	6,450
	CW058	0.33	2.24	0.92	15,735,514	21.51	7,734	0.05	11,367
	CW060	0.35	2.01	0.69	17,420,016	42.73	20,226	0.12	18,946
	CW063	0.3	7.36	0.68	29,563,804	23.9	4,668	0.02	5,334
	CW064	0.31	8.75	0.77	20,965,680	23.99	3,284	0.02	3,339
	CW066	0.31	6.02	6.38	22,995,932	18.01	6,022	0.03	10,477
	CW068	0.36	14.19	0.55	19,904,022	39.97	1,210	0.01	793
	CW070	2.89	33.49	0.57	21,597,408	49.04	1,874	0.01	19
	CW071	0.27	10.6	0.49	13,667,804	30.21	2,444	0.02	2,269
	CW072	2.86	31.83	0.49	17,622,030	49.71	574	0	25
	CW074	0.38	11.43	0.5	9,569,972	23.11	942	0.01	1,190
	CW075	0.28	4.7	0.73	27,074,844	21.49	5,580	0.02	8,983
	CW077	0.33	1.24	0.76	17,411,116	11.59	6,398	0.04	11,753
	CW078	0.33	3.5	1.01	20,981,078	20.33	12,078	0.06	7,790
	CW081	0.82	38.64	1.09	30,980,812	17.7	706	0	240
	CW082	2.23	42.07	0.85	24,518,788	41.96	44,974	0.18	61
	CW084	1.72	150.01	0.6	12,003,682	30.42	580	0.01	41
	CW086	4.13	63.04	0.97	30,820,072	37.55	50,262	0.16	25
	CW087	0.39	43.48	0.9	40,529,896	38.19	6,912	0.02	545
	CW088	0.44	108.91	1	43,215,976	31.26	1,190	0	461
	CW090	0.45	41.81	0.65	29,753,422	23.79	1,216	0	419
	CW093	0.14	20.43	0.78	34,657,992	24.89	4,596	0.01	5,010
	CW094	0.18	18.76	0.73	27,197,300	21.24	4,460	0.02	3,170

	CW095	1.89	59.92	0.77	22,443,570	27.41	18,940	0.08	40
	CW098	0.15	5.63	0.96	36,431,106	15.95	10,396	0.03	18,908
	CW099	8.99	41.59	1.58	33,524,142	54.51	884,588	2.64	25
	CW100	0.3	33.44	0.6	24,861,536	24.22	684	0	343
	CW101	2.85	44.41	0.64	31,999,828	35.52	2,076	0.01	30
	CW102	0.24	41.46	0.68	29,041,550	29.06	6,484	0.02	693
	CW103	0.63	42.83	0.97	30,640,080	34.62	1,016	0	227
	CWp1U	10.5	4.53	1.07	15,197,504	54.85	128,770	0.85	18
	CWp2U	2.25	2.34	1.05	24,161,792	55.65	2,138	0.01	91

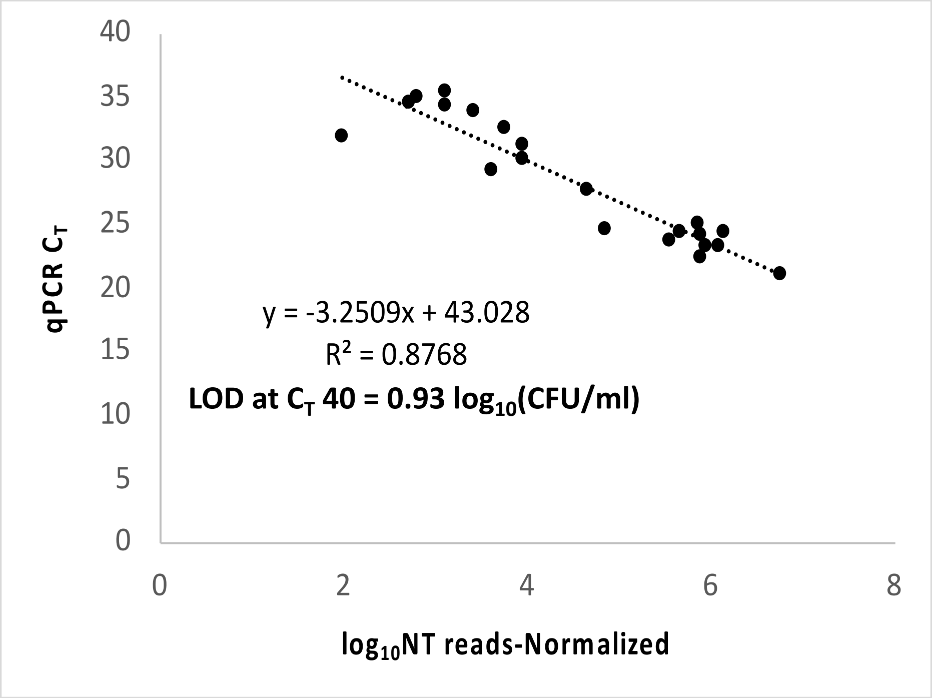

Supplementary Figure 1: Estimated limit of detection (LOD) of mNGS assay for pathogen detection. CSF specimens negative by standard microbiological methods were spiked with a range of viral and bacterial pathogens at varying concentrations as described in the Materials and Methods and simultaneously tested by pathogen specific qPCR and by mNGS as described in the Materials and Methods. mNGS read counts for spiked pathogens were normalized with the genome size (Mbp) of respective pathogens and plotted against qPCR C_T values. Limit of detection was calculated from linear equation at C_T = 40.

Supplementary Figure 2: Heatmap of NGS reads in the training set mapped to pathogen genomes generated in IdSeq by default.

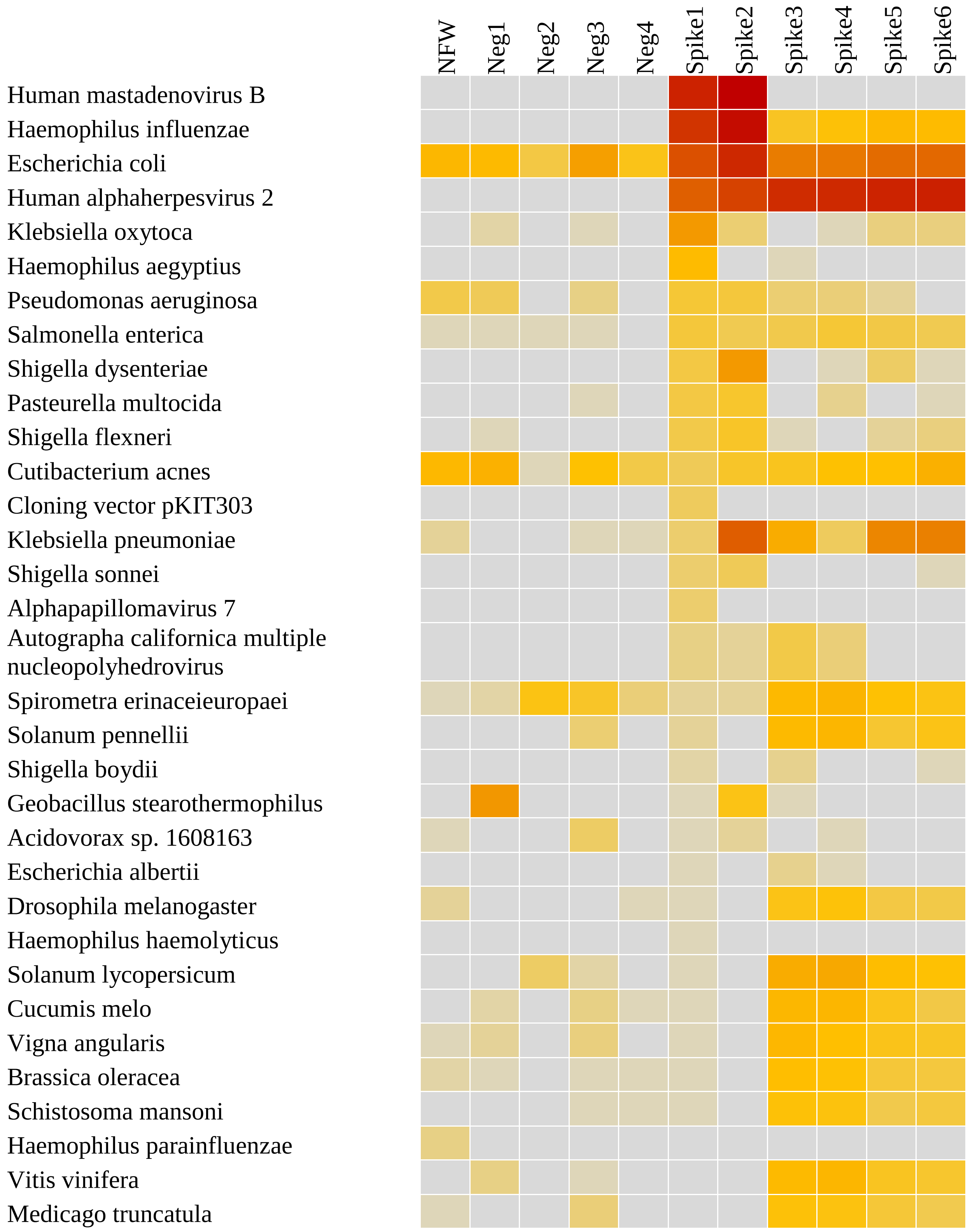

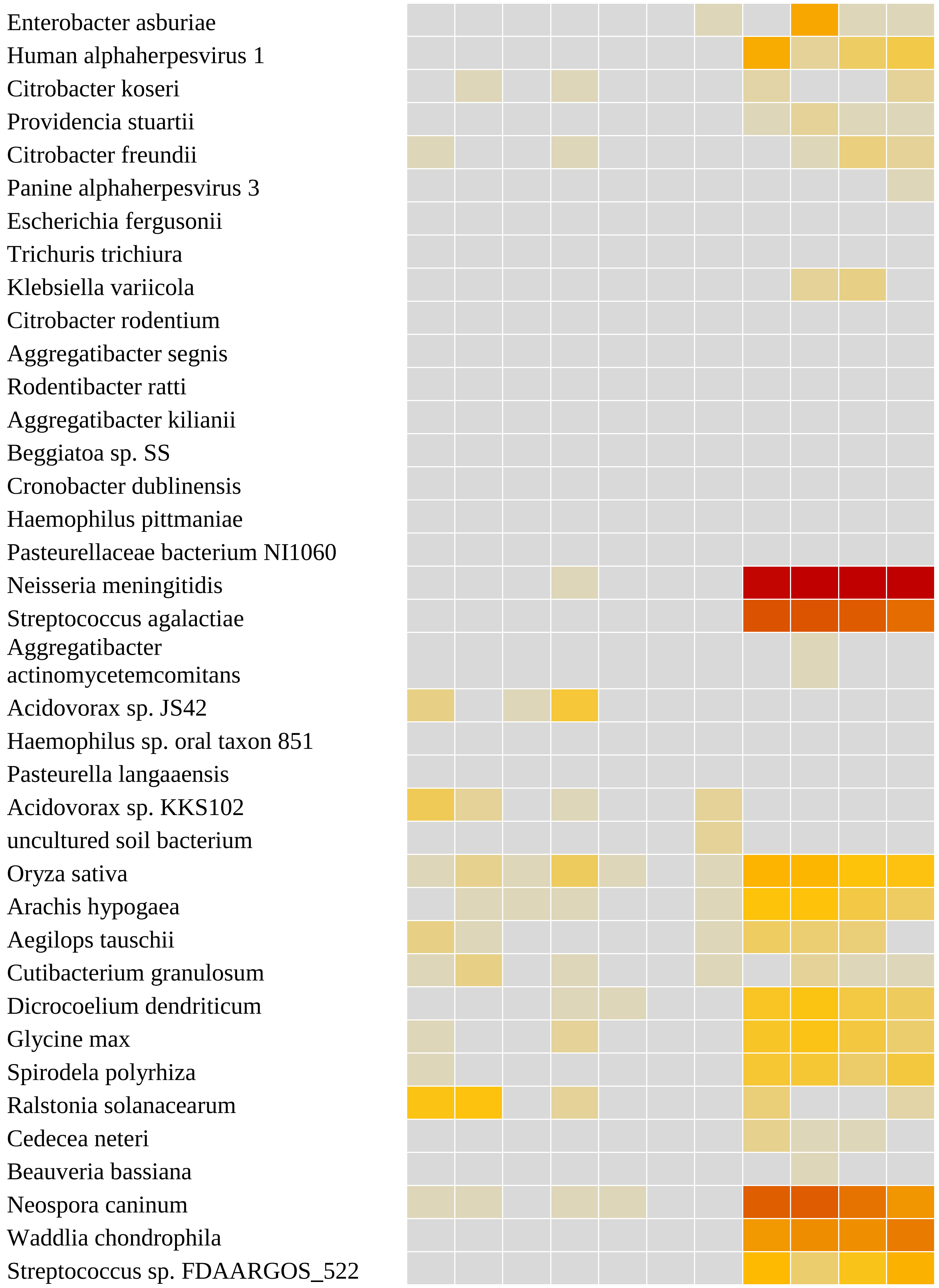
Enterobacter asburiae
Human alphaherpesvirus 1
Citrobacter koseri
Providencia stuartii
Citrobacter freundii
Panine alphaherpesvirus 3
Escherichia fergusonii
Trichuris trichiura
Klebsiella variicola
Citrobacter rodentium
Aggregatibacter segnis
Rodentibacter ratti
Aggregatibacter kilianii
Beggiatoa sp. SS
Cronobacter dublinensis
Haemophilus pittmaniae
Pasteurellaceae bacterium NI1060
Neisseria meningitidis
Streptococcus agalactiae
Aggregatibacter actinomycetemcomitans
Acidovorax sp. JS42
Haemophilus sp. oral taxon 851
Pasteurella langaaensis
Acidovorax sp. KKS102
uncultured soil bacterium
Oryza sativa
Arachis hypogaea
Aegilops tauschii
Cutibacterium granulosum
Dicrocoelium dendriticum
Glycine max
Spirodela polyrhiza
Ralstonia solanacearum
Cedecea neteri
Beauveria bassiana
Neospora caninum
Waddlia chondrophila
Streptococcus sp. FDAARGOS_522

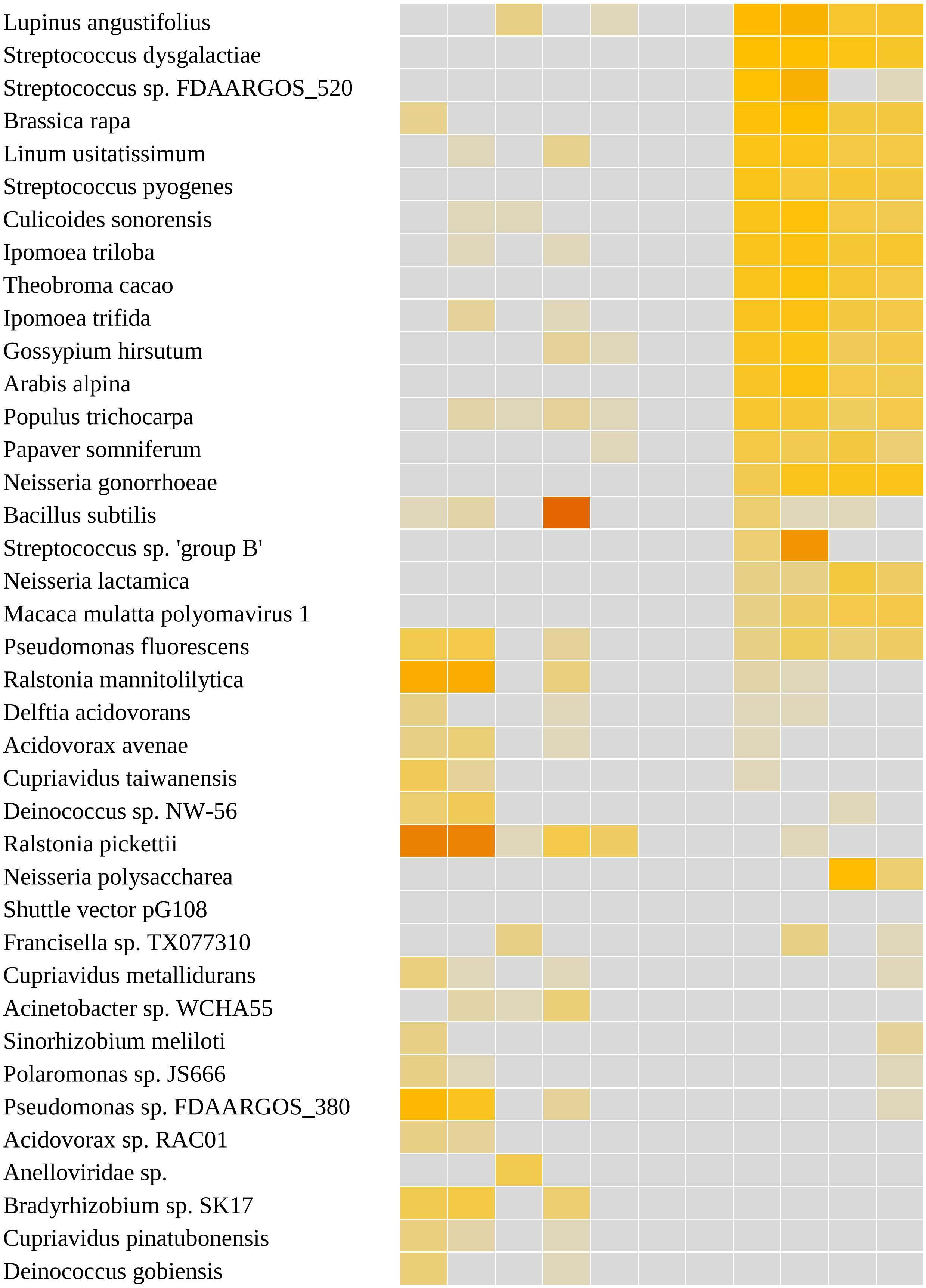
Lupinus angustifolius
Streptococcus dysgalactiae
Streptococcus sp. FDAARGOS_520
Brassica rapa
Linum usitatissimum
Streptococcus pyogenes
Culicoides sonorensis
Ipomoea triloba
Theobroma cacao
Ipomoea trifida
Gossypium hirsutum
Arabis alpina
Populus trichocarpa
Papaver somniferum
Neisseria gonorrhoeae
Bacillus subtilis
Streptococcus sp. 'group B'
Neisseria lactamica
Macaca mulatta polyomavirus 1
Pseudomonas fluorescens
Ralstonia mannitolilytica
Delftia acidovorans
Acidovorax avenae
Cupriavidus taiwanensis
Deinococcus sp. NW-56
Ralstonia pickettii
Neisseria polysaccharea
Shuttle vector pG108
Francisella sp. TX077310
Cupriavidus metallidurans
Acinetobacter sp. WCHA55
Sinorhizobium meliloti
Polaromonas sp. JS666
Pseudomonas sp. FDAARGOS_380
Acidovorax sp. RAC01
Anelloviridae sp.
Bradyrhizobium sp. SK17
Cupriavidus pinatubonensis
Deinococcus gobiensis

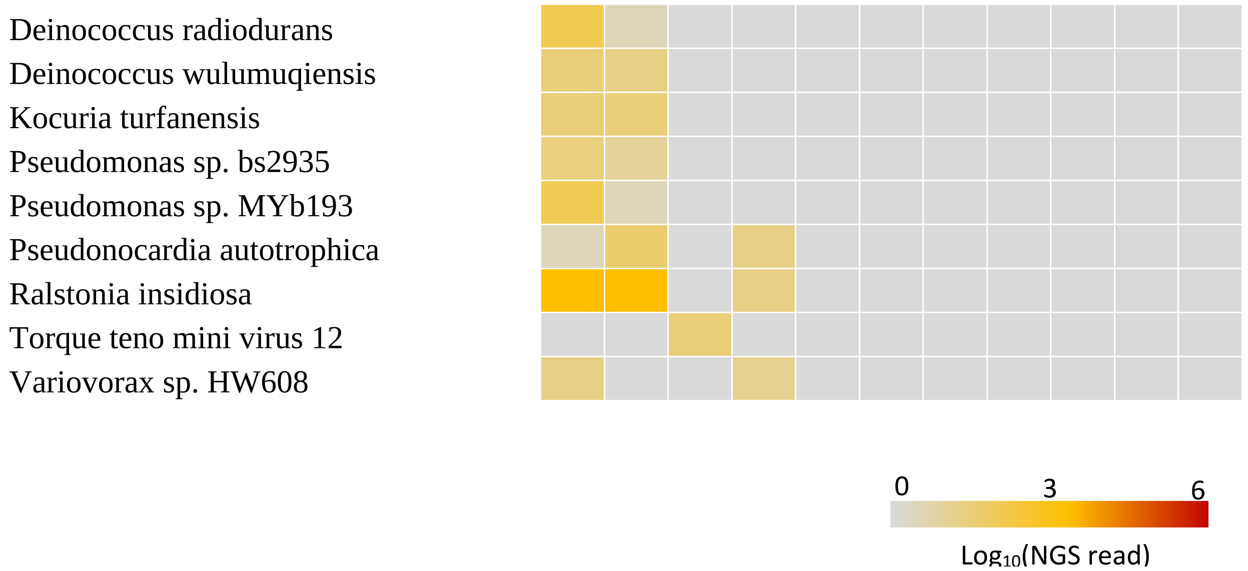
Deinococcus radiodurans
Deinococcus wulumuqiensis
Kocuria turfanensis
Pseudomonas sp. bs2935
Pseudomonas sp. MYb193
Pseudonocardia autotrophica
Ralstonia insidiosa
Torque teno mini virus 12
Variovorax sp. HW608
0
3
6
Log10(NGS read)

Supplementary Figure 3: Heatmap of relative abundance of taxa identified in training set by Metaphlan2

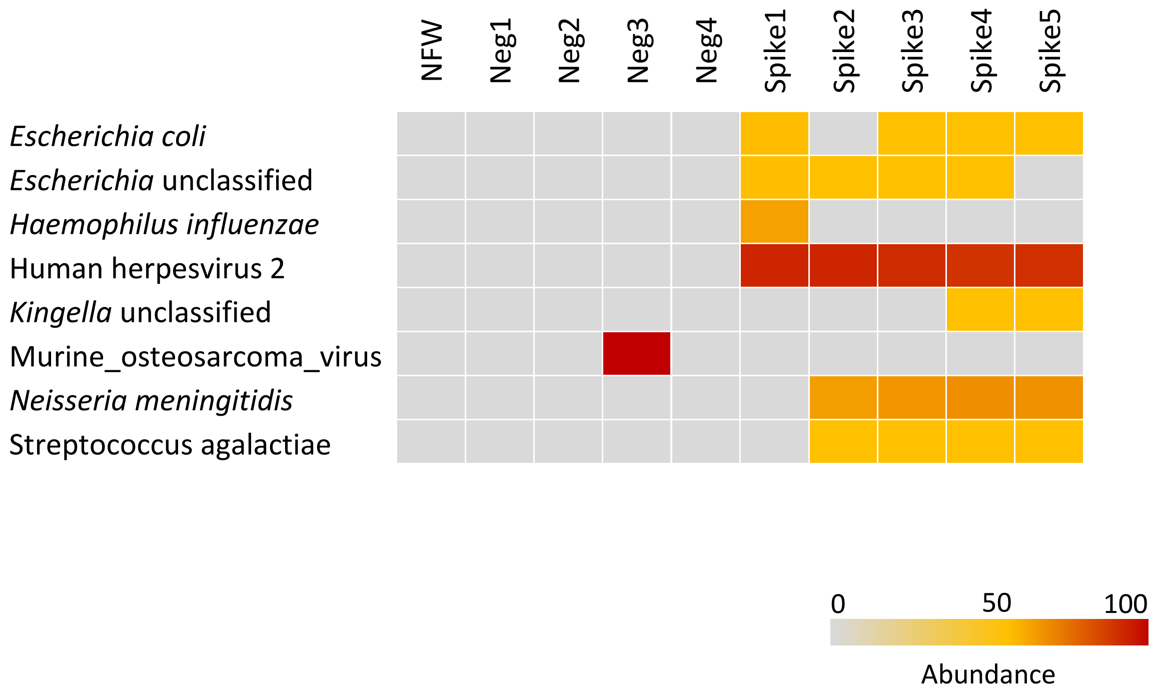

Supplementary Figure 4: Heatmap of NGS reads in the training set mapped to pathogen genomes generated in IdSeq with custom filters.

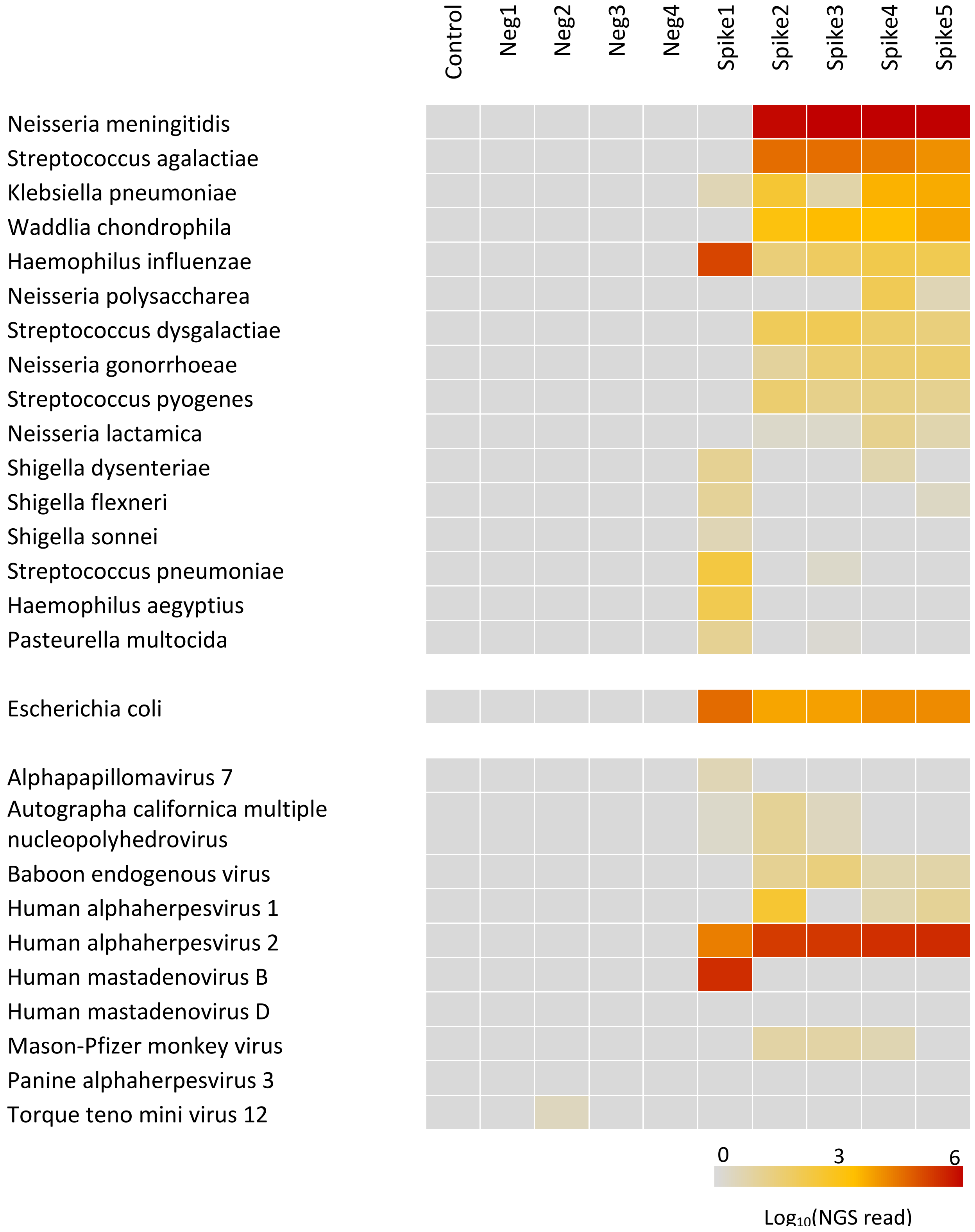

Supplementary Figure 5: Phylogenetic tree of *Escherichia coli* sequence reads in training set specimens

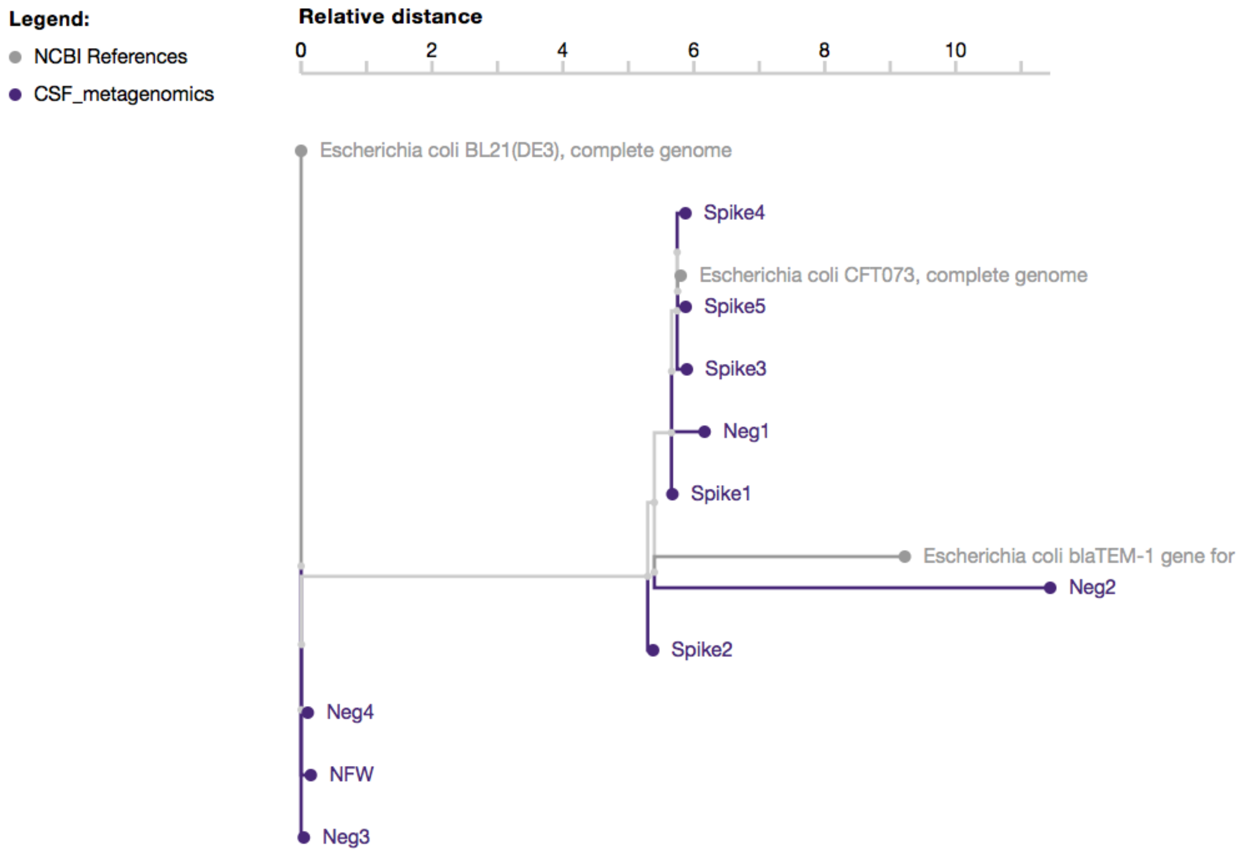

Supplementary Figure 6: Phylogenetic tree of *Escherichia coli* sequence reads in validation set specimens

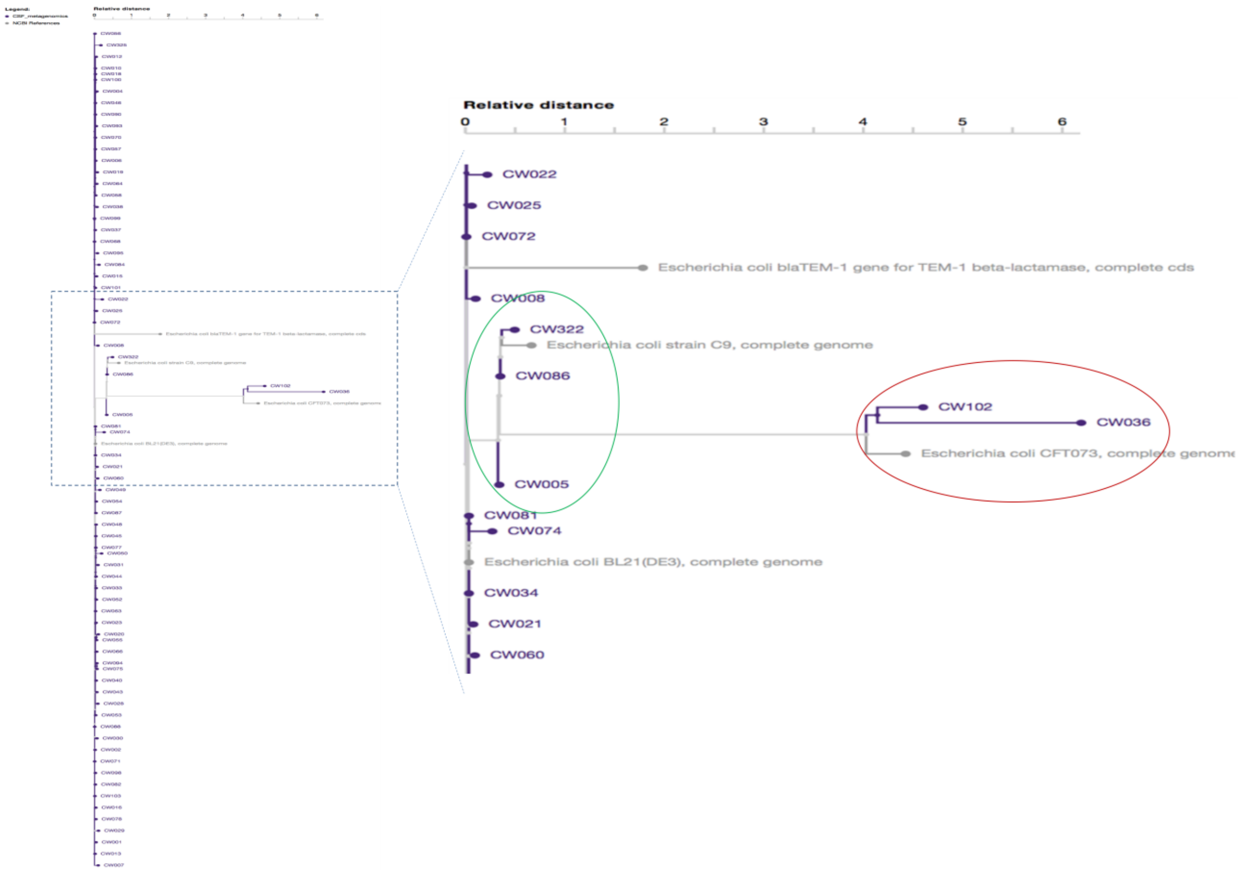

Supplemental table 2: Comparison or mNGS results with conventional and confirmatory test results

Study no.	Gram staining			Culture result	PCR assays performed	PCR result	mNGS result	Result classification	Confirmatory test and result
	WBC	RBC	Org						
CW001	3+	None	None	Negative	HSV1, HSV2, EV, VZV, CMV, EBV	Negative	Negative	True negative	
CW002	2+	1+	None	Negative	HSV1, HSV2, EV	EV	Negative	*True negative	
CW004	1+	2+	None	Negative	HSV1, HSV2, EV, VZV	Negative	Negative	True negative	
CW005	2+	3+	None	Staphylococcus epidermidis reported as contaminant	HSV1, HSV2	Negative	Negative	True negative	Negative by 16s sequencing
CW006	1+	None	None	Negative	HSV1, HSV2, VZV, EBV	Negative	Negative	True negative	
CW007	4+	None	4+ GPC	4+ Streptococcus pneumoniae	HSV1, HSV2, EV, Spn	Spn	Streptococcus pneumoniae	True positive	
CW008	4+	None	None	Negative	HSV1, HSV2, EV, Nmen, Spn	Spn	Streptococcus pneumoniae	True positive	
CW010	2+	None	None	Negative	HSV1, HSV2, EV	Negative	Negative	True negative	
CW012	None	2+	None	Negative	HSV1, HSV2, Spn, EV	EV	Negative	*True negative	
CW013	4+	None	2+ GPC	1+ Streptococcus agalactiae	HSV1, HSV2, EV	Negative	Streptococcus agalactiae	True positive	
CW015	4+	None	None	Negative	HSV1, HSV2, EV, Spn, Nmen, Hflu	Negative	Negative	True negative	
CW016	1+	None	None	Negative	HSV1, HSV21, EV, VZV, EBV, CMV, Spn, Nmen	HSV2	HSV2	True positive	
CW018	1+	4+	None	Negative	HSV1, HSV2, EV, VZV	Negative	Negative	True negative	
CW019	None	None	None	Negative	HSV1, HSV2	Negative	Negative	True negative	
CW020	1+	None	None	Negative	MPN	Negative	Negative	True negative	
CW021	3+	2+	4+ GNCB	3+ Haemophilus influenzae	HSV1, HSV2, EV, EBV	Hflu	Haemophilus influenzae	True positive	
CW022	4+	4+	None	Negative	HSV1, HSV2, EV	EV	Negative	*True negative	
CW023	4+	1+	None	Negative	HSV1, HSV2, VZV, EV, Mpn	Negative	Negative	True negative	
CW024	4+ WBC	None	1+ GPC	Negative	HSV1, HSV2, EV	Negative	Streptococcus agalactiae	False positive	GBS positive by PCR
CW025	1+	1+	None	Negative	HSV1, HSV2, EV, HHV6	Negative	Negative	True negative	
CW028	None	None	None	Negative	HSV1, HSV2, EV	Negative	Negative	True negative	
CW029	3+	None	None	Negative	HSV1, HSV2, EV	Negative	Negative	True negative	
CW030	1+	2+	None	Negative	HSV1, HSV2	Negative	Negative	True negative	
CW031	None	None	None	Negative	HSV1, HSV2, EV, VZV, CMV, HHV6, Mpn, Spn	Negative	Negative	True negative	
CW033	1+	None	None	Negative	HSV1, HSV2, EBV	Negative	Negative	True negative	

CW034	4+ WBC	None	3+ GPC	Negative	HSV1, HSV2, EV	Negative	Streptococcus agalactiae	False positive	GBS positive by PCR
CW036	4+	1+	None	1+ Escherichia coli	Spn	Negative	Escherichia coli	True positive	
CW037	1+	4+	None	2+ Streptococcus agalactiae	HSV1, HSV2, EV	Negative	Streptococcus agalactiae	True positive	
CW038	1+	None	None	Negative	HSV1, HSV2, EV	Negative	Negative	True negative	
CW040	None	None	None	Negative	HSV1, HSV2, EV, VZV, HHV6	Negative	Negative	True negative	
CW043	1+	1+	None	Negative	HSV1, HSV2, EV, VZV, EBV	Negative	Negative	True negative	
CW044	1+	4+	None	Negative	HSV1, HSV2, VZV, EBV	Negative	Negative	True negative	
CW045	1+	1+	None	Negative	HSV1, HSV2, EV	Negative	Negative	True negative	
CW046	1+	3+	None	Negative	HSV1, HSV2, CMV, EBV	Negative	Negative	True negative	
CW048	1+	None	None	Negative	HSV1, HSV2, EV, VZV, HHV6	Negative	Negative	True negative	
CW049	2+	None	None	Negative	HSV1, HSV2	Negative	Negative	True negative	
CW050	None	None	None	Negative	HSV1, HSV2, EV, EBV	Negative	Negative	True negative	
CW052	None	None	None	Negative	HSV1, HSV2, EV, VZV, EBV, Mpn	Negative	Negative	True negative	
CW053	None	None	None	Negative	HSV1, HSV2, VZV, EBV	Negative	Negative	True negative	
CW054	None	None	None	Negative	HSV1, HSV2, EV	Negative	Negative	True negative	
CW055	None	None	None	Negative	HSV1, HSV2, EV	Negative	Negative	True negative	
CW056	2+	1+	None	Negative	HSV1, HSV2, EV	Negative	Negative	True negative	
CW057	1+	None	None	Negative	HSV1, HSV2, VZV, EV	Negative	Negative	True negative	
CW058	None	None	None	Negative	HSV1, HSV2, EV, VZV	Negative	Negative	True negative	
CW060	None	None	None	Negative	HSV1, HSV2, VZV	Negative	Streptococcus parasanguinis	False positive	Negative by 16s sequencing
CW063	None	None	None	Negative	HSV1, HSV2, EV, VZV	Negative	Negative	True negative	
CW064	1+	1+	None	Negative	HSV1, HSV2, EV	Negative	Negative	True negative	
CW066	None	None	None	Negative	HSV1, HSV2, EV, VZV	Negative	Negative	True negative	
CW068	1+	1+	None	Negative	HSV1, HSV2, EV	EV	Negative	*True negative	
CW070	3+	1+	None	Negative	HSV1, HSV2, EV, Spn	Spn	Streptococcus pneumoniae	True positive	
CW071	1+	None	None	Negative	HSV1, HSV2, VZV, EV	EV	Negative	*True negative	
CW072	4+	4+	None	Negative	HSV1, HSV2, VZV, HHV6, EV	EV	Negative	True negative	
CW074	None	None	None	Negative	HSV1, HSV2, EV	Negative	Negative	True negative	
CW075	1+	None	None	Negative	HSV1, HSV2, EV	Negative	Negative	True negative	
CW077	None	None	None	Negative	HSV1, HSV2, VZV, EBV, EV	Negative	Negative	True negative	
CW078	None	None	None	Negative	HSV1, HSV2, EV, VZV	Negative	Negative	True negative	

CW081	3+	None	None	Negative	HSV1, HSV2, EV, EBV, Spn	Negative	Negative	True negative	
CW082	4+	None	4+ GPC	4+ Streptococcus agalactiae	HSV1, HSV2, EV, GBS	GBS	Streptococcus agalactiae	True positive	
CW084	2+	4+	None	Negative	HSV1, HSV2, EV	EV	Negative	*True negative	
CW086	None	None	None	Negative	Spn, Hflu, Nmen	Nmen	Neisseria meningitidis	True positive	
CW087	4+	4+	None	Negative	HSV1, HSV2, EV, Nmen, Spn	Nmen	Neisseria meningitidis	True positive	
CW088	2+	4+	None	Negative	HSV1, HSV2, EV	EV	Negative	*True negative	
CW090	1+	2+	None	Negative	HSV1, HSV2, EV, EBV	EV, EBV	EBV	True positive	EBV positive by PCR
CW093	None	None	None	Negative	HSV1, HSV2, EV, VZV, HHV6	Negative	Negative	True negative	
CW094	2+	None	None	Negative	HSV1, HSV2, EV, VZV	Negative	Negative	True negative	
CW095	4+	4+	None	1+ Streptococcus agalactiae	HSV1, HSV2, EV, VZV, CMV, EBV, HHV6, Spn	Negative	Streptococcus agalactiae	True positive	
CW098	1+	4+	None	Negative	HSV1, HSV2, EV, VZV	Negative	Negative	True negative	
CW099	4+	2+	4+ GPC	4+ Streptococcus pneumoniae	HSV1, HSV2, EV, CMV	Negative	Streptococcus pneumoniae	True positive	
CW100	4+	None	None	Negative	Spn	Negative	Negative	True negative	
CW101	1+	4+	None	1+ Candida tropicalis	HSV1, HSV2, EV, CMV	Negative	Candida tropicalis	True positive	Candida tropicalis by ITS sequencing
CW102	4+	4+	1+ GNC	2+ Escherichia coli	Nmen, Spn	Negative	Escherichia coli	True positive	
CW103	2+	1+	None	Negative	HSV1, HSV2, EV, VZV, Mpn	Negative	Negative	True negative	
CW322	4+	None	None	1 colony Roseomonas species, 1 colony Staphylococcus epidermidis; reported as contaminants	Spn	Nmen	Neisseria meningitidis, **HSV2	True positive	
CW325	1+	4+	None	Negative	HSV1, HSV2	HSV2	HSV2	True positive	

*True negative for validation purposes only because the mNGS assay described in this study is not expected to detect RNA viruses

**HSV2 result could not be confirmed because no left over sample was available

HSV1, herpes simplex virus 1; HSV2, herpes simplex virus 2; EV, enterovirus; VZV, varicella zoster virus; CMV, cytomegalovirus; EBV, Epstein-Barr virus; HHV6, human herpes virus 6; Spn, *Streptococcus pneumoniae*; Mpn, *Mycoplasma pneumoniae*; Nmen, *Neisseria meningitidis*; GBS, Group B *Streptococcus* or *Streptococcus agalactiae*
